# Steroids and Brain, a Rising Bio-Medical Domain: a Perspective

**DOI:** 10.3389/fendo.2018.00316

**Published:** 2018-06-15

**Authors:** Etienne-Emile Baulieu

**Affiliations:** INSERM UMR 1195, Université Paris-Saclay, Hôpital Le Kremlin Bicêtre, Val de Marne, France

**Keywords:** brain steroids, neurosteroids, Alzheimer’s disease, tauopathies, pregnenolone, MAP4343, FKBP52, RU486

## Abstract

Some newly described steroid-related compounds, also found in the rest of the body, are formed and active in the central nervous system, particularly in the brain. Some are of pharmacological and physiopathological interest. We specifically report on two compounds, “MAP4343,” a new neurosteroid very efficient antidepressant, and “FKBP52,” a protein component of hetero-oligomeric steroid receptors that we found involved in cerebral function, including in Alzheimer’s disease.

## Introduction

**Figure 1:**
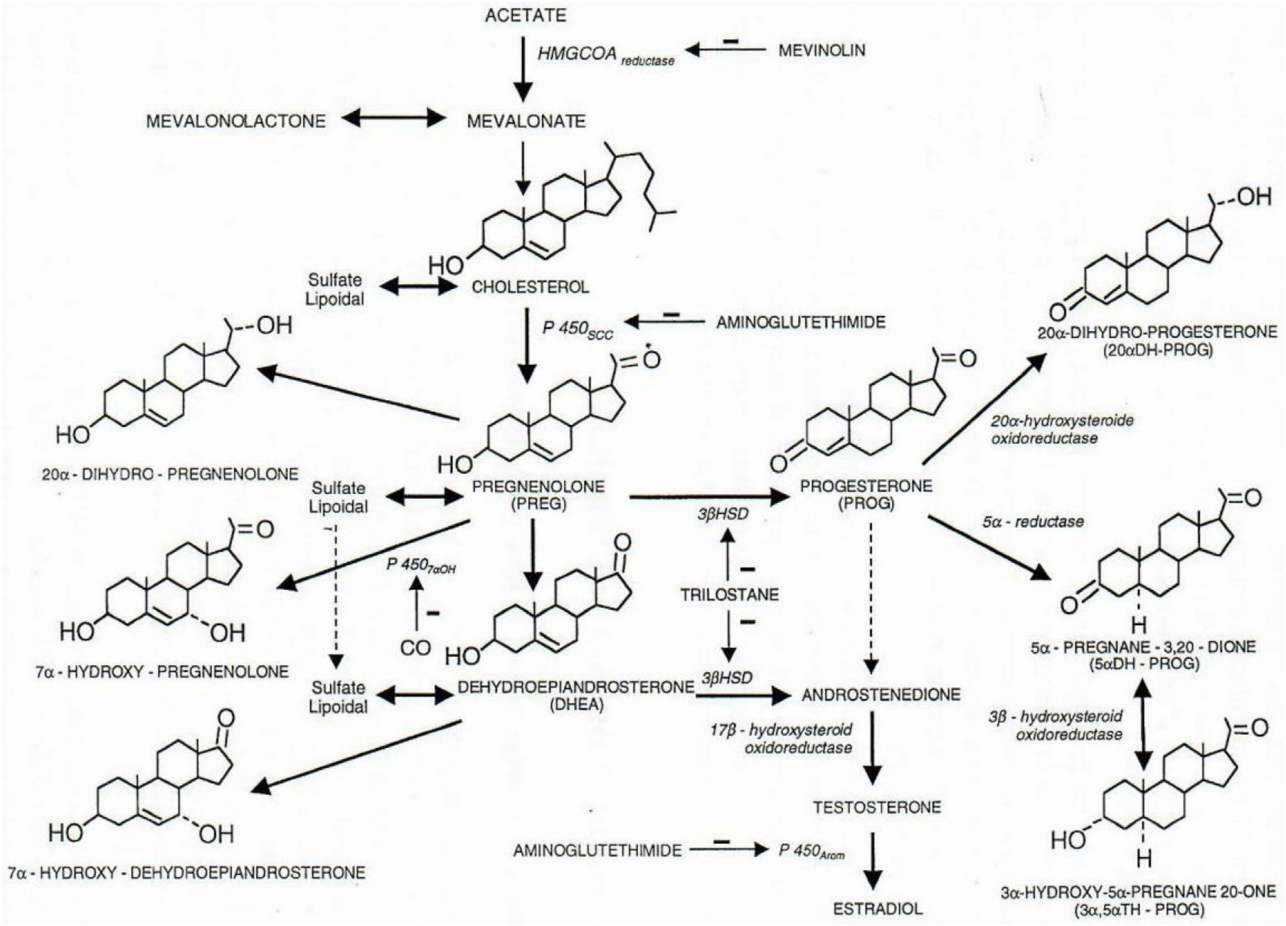
Some metabolic pathways of physiological steroids. All steroids are cholesterol derivatives. The P450scc cytochrome (scc for side chain cleavage) cut the 6 carbon chain of cholesterol, and pregnenolone thus synthetized is precursor of all steroid hormones (3β-HSD, 3β-hydroxysteroid dehydrogenase; 3α-HSOR, 3α-hydroxysteroid oxidoreductase; 17β-HSOR, 17β-hydroxysteroid oxidoreductase). Negative signs (−) indicate the lack of the enzymatic function corresponding to the indicated product. Figure from Baulieu (5).

Some cholesterol found in brain and spinal cord is biologically largely independent of that found in the rest of the body (Figure 1). It gives rise to “neurosteroids” (1) and we mention in this short “Perspective” some of their pharmacological and pathophysiological properties. In particular, we report the therapeutic antidepressant activity of a new neurosteroid drug “MAP4343,” derivative of pregnenolone (PREG) (2). Very differently, we mention the cloning (3) and the function of a protein, “FKBP52,” which is a component of hetero-oligomeric steroid receptors and that, with Chambraud et al. (4), we found involved in the function of the cerebral Tau protein, including in Alzheimer’s disease. Thus, we take care of two novel and distinct active steroid-related compounds of medical interest.

**Figure 2:**
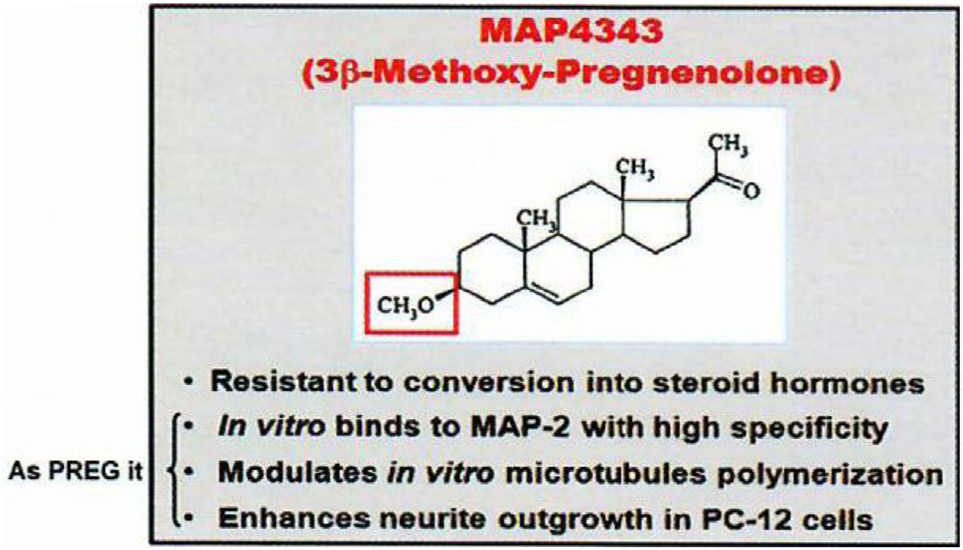
MAP4343, a pregnenolone derivative. Data from Baulieu (8).

First, among “neurosteroids” (1), PREG (6) is rather important quantitatively, and MAP4343, its synthetic derivative 3β-methoxy-Δ5-pregnene-20 one [Figure 2; (7)], is remarkably active to treat depressive states and addiction to ethanol (in preparation with G. Koob and O. George); it is currently studied in human beings.

Second, “FKBP52” is a protein complexed with hetero-oligomeric steroid receptors that we found to have an unforeseen interaction (4) with the Tau protein centrally involved in Alzheimer’s disease and other dementias. The profound decrease of FKBP52 in several tauopathies (9) suggests its possible therapeutic importance.

## Neurosteroids: MAP4343, A Therapeutically Active Derivative of PREG

The synthesis of cholesterol in the nervous system has been described by Bloch (10). Its metabolic steroids synthetized in the central and the peripheral nervous systems (11, 12) are called neurosteroids (1, 13); their syntheses in vertebrate brains (14) occurs in neurons and glial cells, sometimes from some imported steroidal precursors. Neurosteroids include several 3β-hydroxy-Δ5 compounds, such as PREG (6, 15) and dehydroepiandrosterone (DHEA) (16–19), and their sulfate esters (20, 21). Moreover, there are Δ4-3oxo steroid hormones (progesterone is quite abundant) (22), and some of their reduced metabolites, such as one of the tetrahydroderivatives of progesterone (3αhydroxy-5αpregnan-20one, also called allopregnanolone) (23, 24). Several neurosteroids can act as modulators of neurotransmitter receptors, in particular those of GABA_A_ (25), NMDA, and sigma-1. Frequently, 3β-hydroxy-Δ5 steroids are metabolized to Δ4-3oxo steroids in brain by 3βHSD (3β-hydroxysteroid dehydrogenase). Δ4-3oxo neurosteroids act *via* classical nuclear steroid receptors (26). Recent studies with progesterone indicate the possibility of distinct hormonal derivatives (27). 3β-hydroxy-Δ5 compounds themselves can also bind to the microtubule-associated protein MAP2 (28). With different locations through the brain, neurosteroids may have several concentrations and/or display diverse activities with various roles on environment and behavior (5).

The oxidative transformation of PREG to progesterone and the metabolism of progesterone to other active neurosteroids (including androgens and corticosteroids) have led us to avoid the metabolism in the brain of 3β-hydroxy-Δ5 neurosteroids to oxydated 3oxo compounds; for this reason, we synthetized and used 3β-methoxy-Δ5 steroids which are not metabolizable to Δ4-3oxo steroids of unnecessary or even pathogenically activity: for instance, an appropriate synthetic derivative of PREG is the methoxylated compound MAP4343 [Figure 2; (7)]. Interestingly, both PREG and its 3β-methoxy derivative can bind to protein MAP2 and they display the same activity on the microtubular system through their association to this protein (29). The interaction of MAP2 with PREG (natural neurosteroid) or MAP4343 (synthetic derivative) modifies the function of microtubules in target cells, stimulates their assembly, and is then responsible of positive improvement of behavior: not metabolizable to Δ4-3oxo steroids contrarily to PREG (which therefore cannot be safely administered as such). MAP4343 is very active and therefore medically convenient. Experimentally, MAP4343 increases anxiolytic and anti-depressive activities (30) in rats submitted to a specific psychosocial condition (31), in stressed tree shrews (32), and in Kyoto rats resistant to currently available antidepressants (to be submitted by Villey et al. in 2018). MAP4343 is therapeutically rapid and remarkably safe.

There are other results making 3βhydroxy-Δ5 steroids interesting to study. Their deficit in hippocampus of aged rodents may be responsible for some cognitive alteration (33); however, a similar effect has not yet been demonstrated in human beings. It is also worthwhile to analyze the possible effect of cerebral DHEA and its sulfate (34), unevenly distributed in the human brain.

**Figure 3:**
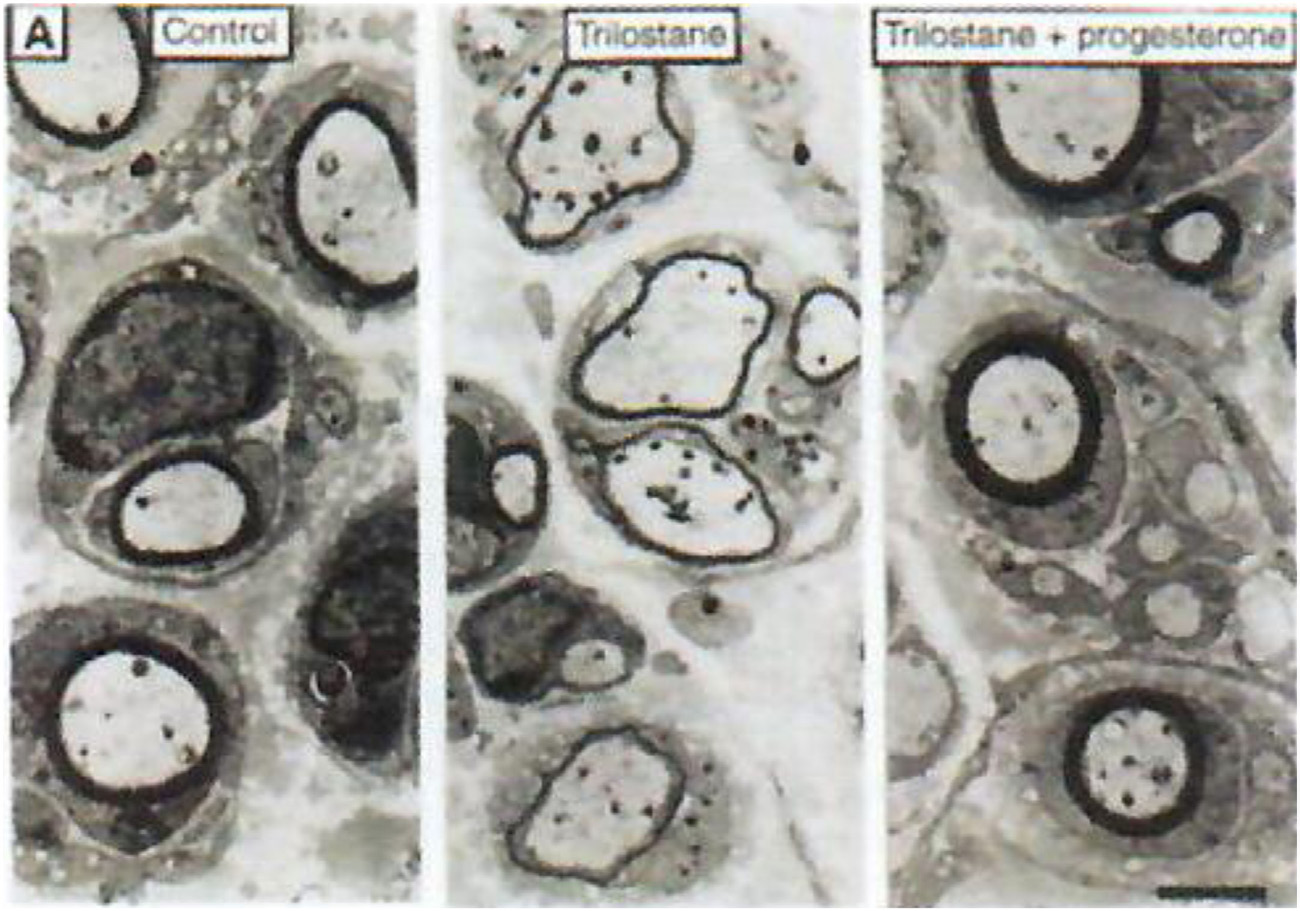
Role of neurosteroids in the formation of myelin sheaths. The thickness of myelin sheaths (number of lamellae) was quantified by electron microscopy of cross section of sciatic nerves from male mice 15 days after cryolesion. Effect of trilostane in the absence (center panel) or presence (right panel) of progesterone on the thickness of myelin sheaths, relative to that in control nerves (left panel). Scale bar, 2 µm. Data from Koenig et al. (38).

**Figure 4:**
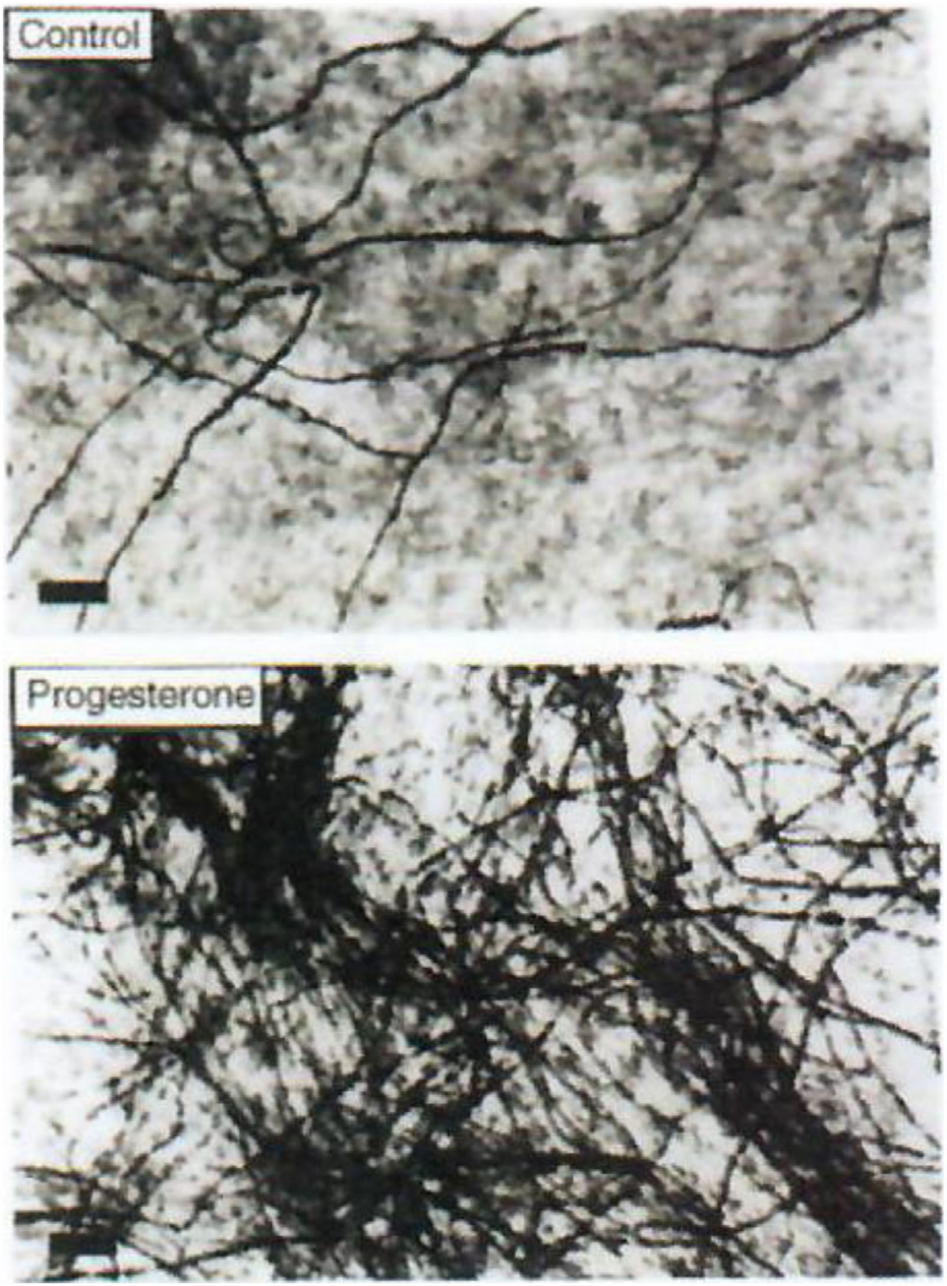
Effect of progesterone on myelin formation in DRG cultures. Cells were cultured for 2 weeks in myelination-promoting medium in the absence (top) or presence (bottom) of 20 nM progesterone. Myelinated fibers were stained with Sudan black and the number of myelin segments was determined. Only myelinated fibers are visible on these photographs. Scale bars, 40 µm. Data from Koenig et al. (38).

Progesterone is a steroid difficult to study in the central nervous system (CNS) both qualitatively and metabolically. Progesterone is particularly involved in protection/repair after traumatisms of the CNS (35–37) and it plays a fundamental role in (re)myelination (Figures 3 and 4). After cryolesion of peripheral nerves, we have analyzed the function of progesterone synthetized by Schwann cells (38, 39).

A particular transcription factor “Krox-20,” expressed in Schwann cells and stimulated by progesterone, plays an important role in myelination of regenerating sciatic nerve and sensory neurons (40, 41). The blockade of progesterone stimulation by RU486 suggests a function as for a classical progesterone receptor. There is also another membrane-associated progesterone-binding protein initially called 25Dx and currently known as PGRMC [progesterone receptor membrane compound; (42)].

Moreover, besides genomic mechanism, membrane actions of progesterone in the CNS have been described (27). Several membrane progesterone receptor(s) have been discovered (27, 43–47). It is also interesting to note that the reduced tetrahydro-metabolite of progesterone (allopregnanolone), at nanomolar concentration, can modify GABA evoked current (45). Several other neurosteroids modify functionally the activity of neuromediator receptors.

It is clear that the pharmacological function of some neurosteroids such as progesterone is diversified and this may be the reason for which it is not easy to rationalize the medical usage of post-menopausal hormone replacement (48). In addition, there are a number of synthetic progesterone analogs, multiplying the variety of compounds displaying some selective function(s) of progestins (49).

There are also a number of different neuroestrogens which, as estradiol (50), include a phenolic nucleus equivalent to the Δ4-3oxo structure of other steroid hormones and have distinct metabolic properties (51).

Δ4-3oxo neuroandrogens, the best known and active being testosterone, are partly derived from neurosteroidal 3β-hydroxy-Δ5 compounds such as DHEA, but androgenic neurosteroids synthetized in the brain have not yet been properly quantified. The androgen receptor is decisive in the spontaneous regeneration of myelin (52).

It is not yet known if, in the brain, there are neurosteroids with glucocorticosteroid properties active by themselves, or if there are, in the brain, only corticosteroids transferred from the body and participating to the quantitative regulation of their own production.

In summary, not only PREG, DHEA, as well as their sulfates, may by themselves be active in the brain, they also are potential precursors in the CNS of Δ4-3oxo steroids which are biologically active *via* binding to nuclear receptors. There are also, making it even more varied, well-known hormonal steroids certainly imported at least in part into the brain from glands of the body which have a function of physiologically quantitative importance on the development and the activities of neurons and myelination. They are not really “neurosteroids,” even if they are intimately linked to the function of the CNS.

### Other Pharmacological Compounds With Activities Related to Neurosteroids

#### An Enantiomeric Form of PREG Sulfate (53–55)

This synthetic compound (56) is curiously much promnesic than PREG sulfate itself, and this effect opens a new field of research to compare activity between natural and enantiomeric structures of hormonal steroids [parenthetically, the natural PREG and PREG sulfate both are themselves active on memory; (21)].

#### Lithium

It has neuroprotective activity, particularly for remyelination of peripheral nerves (57). The results open perspectives in treatments by an inhibitor of glycogen synthase kinase 3β such as lithium.

#### Etifoxine (58, 59)

This neurostimulant of estrogens and progestins is active on experimental autoimmune encephalomyelitis, a model of multiple sclerosis (60, 61). However, attempts to decrease post-partum relapses in sick women (62) have not shown a significant success.

#### RU486 (Mifepristone, an Efficient Anti-Progesterone) (63)

The compound, which is also antiglucocorticosteroid, can be orally administered and has demonstrated anti-neurosteroidal effects on inappropriate neuroprogesterone and neuroglucocorticosteroid pathologies. It also permits protective effects against traumatic neuronal alterations [for example, protection of cerebellar Purkinje cells (64, 65)] and has shown therapeutically activity on some psychotic depressions and meningiomas (unpublished).

## Alzheimer’s Disease: FKBP52, A Constituent of Hetero-Oligomeric Steroid Hormone Receptors, Interacts with TAU Structure and Function

In human beings, the most frequent and severe human senile dementia, the Alzheimer’s disease, currently is not biochemically explained nor treated in order to recover. However, there are several recent progresses for the diagnostic, biomarkers and imagery of the sick brain (66). Since the publication of Alois Alzheimer in 1907, two proteins remain of greatest interest (67). Up to now, the most studied has been “amyloid-β” (Aβ), essentially observed between neurons but its study has still not been successful in any therapeutic approach. The other protein, the structure and the cloning of which have been only precisely analyzed since the 1980 period, is “Tau” (68–70). Besides association to microtubules, the Tau protein is involved in several other cellular functions, including gene regulation (71). In human patients, Tau is present within neuronal cells, and the six isoforms, hyperphosphorylated (72) according to a well-defined pattern (73), are also partly truncated by caspase activity (74). Isomerization and oligomerization of Tau due to FKBP52 are independent processes (75). These modifications are largely responsible for fibrillation and aggregation, and they contribute to establish intracellular neurodegeneration in transgenic animals. It is accepted that modified Tau can be responsible for altered function of nerves, even if details of the mechanism of pathological function of abnormal Tau are not well explained.

Distinct from Alzheimer’s disease, there are dementias which are exclusively tauopathy (69), supporting the belief that Tau abnormalities are directly responsible of dementia activity of the brain in Alzheimer’s disease (72); the tauopathic diseases without Aβ abnormality could be called “pure tauopathies”: some are FTDP17: frontotemporal dementias and parkinsonism linked to chromosome 17; progressive supra-nuclear palsy; Pick diseases, etc.

Consequently, Tau, mostly in aggregated forms, is clearly involved principally in terms of neurodegeneration, and pathological Tau may be most appropriate as potential target for treating Alzheimer (76). The pure tauopathies, human and experimental in animals, are very severe diseases and matters of a large number of studies: in particular, the diseases due to P301L mutation of Tau have been very much studied as convenient models. In genetically P301L mutated zebrafishes, the Tau is much oligomerized and abnormally phosphorylated (77).

**Figure 5:**
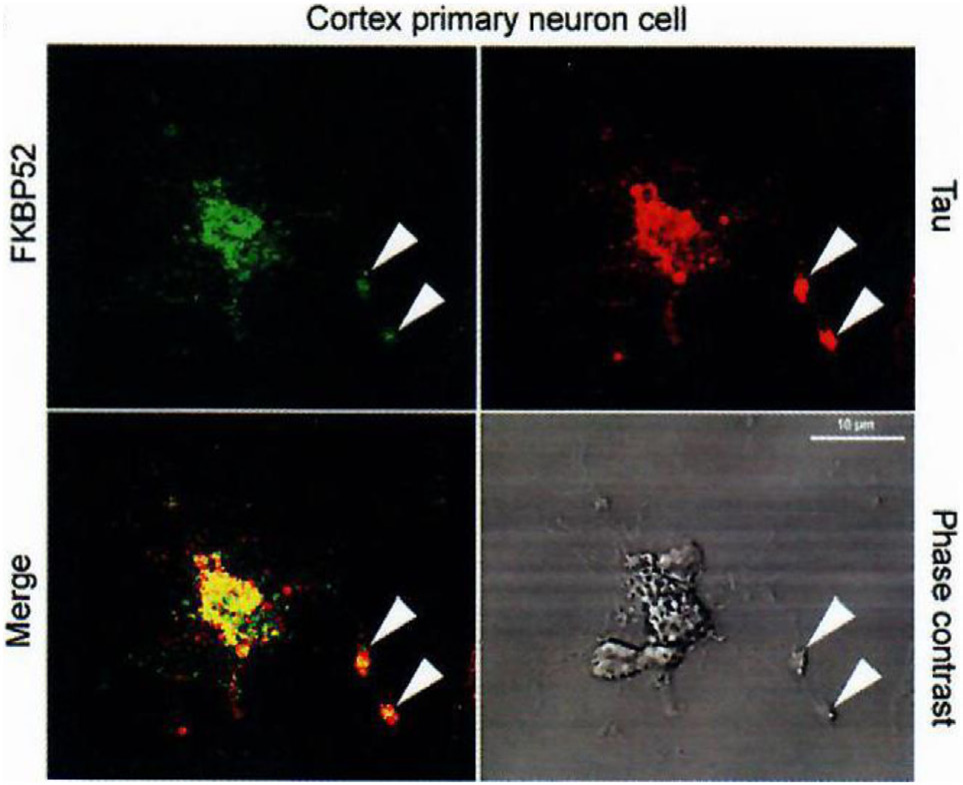
Colocalization of FKBP52 and Tau in primary cortical neurons and PC12 cells. Immunofluorescence staining of primary cortical neurons and PC12 cells treated with 50 nM NGF for 5 days. Double staining for Tau and FKBP52 was performed after cytosol extraction to reveal cytoskeletal association. Arrows indicate preferential colocalization of both proteins in the distal part of the nerve cell axon and at the extremity of PC12 cell neurites. Confocal images of primary cortical neurons. Analysis of 0.5-µm slices confirms the preferential colocalization in the distal part of the axon (arrowheads). Data from Chambraud et al. (4).

In studying steroid receptors in human beings, we had analyzed some of their hetero-oligomeric variants (78, 79). They differ according to diseases, but often include the protein FKBP52. In Alzheimer’s disease, this protein colocalizes with the autophagic-endolysosomal system (Figure 5). The FKBP structure includes an N-terminal domain (FK1) which harbors a sequence with enzymatic PPIase activity (peptidyl-*cis*/*trans*-prolyl-isomerase), thus classifying FKBP52 among the immunophilins which may bind many small molecular ligands (80, 81). The second domain of FKBP52, FK2, is architecturally similar to the FK1 but does not have a PPIase activity. The rest of the FKBP52 protein includes three TetratricoPeptid Repeats’ (TPR) sequences and binds to hsp90, which itself interacts with an hormonal steroid molecule, completing the hetero-oligomeric composition of steroid receptors. After TPR, there is a C terminal sector-binding calmodulin. It is therefore *via* FKBP52 that the hetero-oligomeric steroid hormones could interact functionally with a neuro-protein such as Tau.

**Figure 6:**
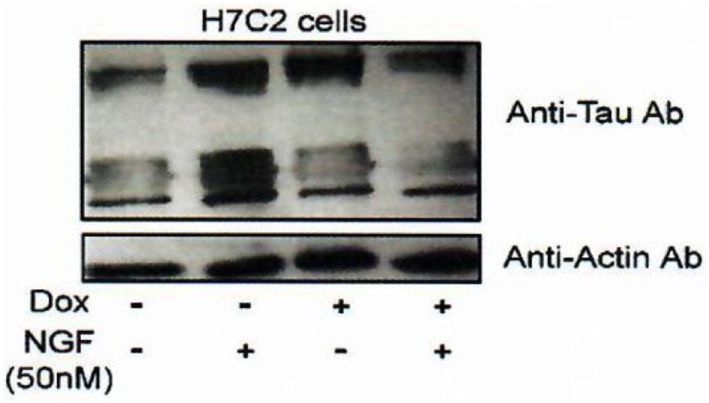
Effect of FKBP52 overexpression on Tau protein accumulation. H7C2 cells: stably transformed PC12 cells by an FKBP52-inducible expression system based on a tetracycline responsive element (82). NGF: 5 days—Dox: 7 days. H7C2 and PC12 cells were treated or not with NGF for 5 days in the presence or absence of DOX for 1 week; 50 µg of extracts was subjected to SDS-PAGE and immunoblotted with anti-Tau (antibody clone DC25). Actin was used as the loading control. Data from Chambraud et al. (4).

Indeed, we had, additionally and very importantly, discovered an interaction of FKBP52 with Tau [(4); Figures 5 and 6], completing the description of steroid–FKBP52–Tau sequence(s) never described till now, and that have not been studied in functional terms.

Experiments with zebrafishes under the direction of Dr. Marcel Tawk (77) utilizing transgenic animals expressing the human Tau P301L, severely suffering of abnormal escape behavior and with some change of Tau phosphorylation, indicated, *in vivo* and *in vitro* studies, the functional abnormalities due to the pathogenic mutation of Tau.

**Figure 7:**
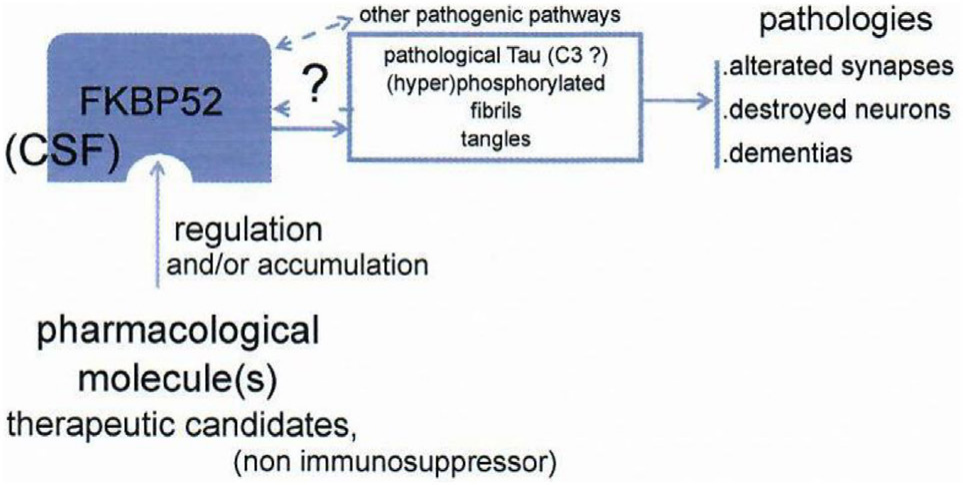
Schema of the working hypothesis and strategy for prevention/treatment of dementias *via* the novel target FKBP52. FKBP52 is measurable in CSF. Data from Baulieu (8).

This is the basis of a strategy leading to look for a treatment of tauopathies, including Alzheimer’s disease and other dementias. FKBP52 may serve for inducing qualitatively and anti-pathological Tau activity and/or directly decreases Tau: considering that Tau is centrally involved in the pathophysiology of Alzheimer, we look for modifying the function of FKBP52 to obtain an actively anti-pathological Tau effect. There is a very profound decrease and/or a reduced function of FKBP52 that we have described in pathological circumstances: in Alzheimer’s disease, FTDP17 (9), and other tauopathies. Our hypothesis is based on the concept that the function of Tau is decisively pathological in dementias, and we hypothesize a FKBP 52 induced change quantitatively and/or qualitatively, which could be profitable to the patients (Figure 7) treated as early as possible after the beginning of the disease. In summary, appropriate modification of FKBP52 induced by some ligand(s) may help to normalize Tau activity and consequently be able to improve the cerebral function.

It is not excluded that several brain steroids may modify the function of parameters implied in this novel approach for treating cerebral abnormality. Other approaches may help to understand alterations of the Tau protein: for instance, studies of synaptic structure (83, 84) and activity (in preparation), and experiments of deep cerebral stimulation can be involved in methods including autophagic-lysosomal protection (85).

Our therapeutic approach centered on effect of FKBP52 on Tau (dys)function may be still far to be operationally favorable to treat dementias (Figure 7). Working on Tau with new modified/liganded FKBP52 function and steroid activities, we will obtain novel results. Will they be valuable?

## Summary

In this “Perspective,” we have treated two distinct examples concerning steroid associated molecules involved in activities of the CNS. They exemplify typical compounds of (1) neurosteroid derivatives and (2) components of hetero-oligomeric steroid receptors. We are happy that, in a number of other laboratories, many publications (not reported in this “Perspective”), have followed our two original studies. This paper is not a review, but we hope to draw attention to the versatile involvement of the steroidal structure and thus in important physiopathological derivatives.

## Author Contributions

The author confirms being the sole contributor of this work and approved it for publication.

## Conflict of Interest Statement

The author declares that the research was conducted in the absence of any commercial or financial relationships that could be construed as a potential conflict of interest.
